# Supplementary material for: Accurate global and local 3D alignment of cryo-EM density maps using local spatial structural features
Source: Nat Commun. 2024 Feb 21;15:1593. doi: 10.1038/s41467-024-45861-4 (PMC10881975; doi:10.1038/s41467-024-45861-4)
Supplement: Supplementary file 1 — Supplementary Information [file 41467_2024_45861_MOESM1_ESM.pdf]

Accurate global and local 3D alignment of cryo-EM density maps using local spatial structural features

Bintao He[1,†], Fa Zhang[2,†], Chenjie Feng[3], Jianyi Yang[1], Xin Gao[4,*], Renmin Han[1,*]

[1]Research Center for Mathematics and Interdisciplinary Sciences, Shandong University, Qingdao 266237, China; [2]School of Medical Technology, Beijing Institute of Technology, Beijing 100081, China; [3]College of Medical Information and Engineering, Ningxia Medical University, Yinchuan 750004, China; [4]King Abdullah University of Science and Technology (KAUST), Computational Bioscience Research Center (CBRC), Computer, Electrical and Mathematical Sciences and Engineering (CEMSE) Division, Thuwal, 23955, Saudi Arabia.

S1 Local spatial feature descriptors

In point cloud registration, surface normals are commonly used as attributes for individual points. They provide valuable information about the local surface orientation. Many effective feature descriptors have been proposed by collecting information about the distribution of normals in local regions (Salti *et al.*, 2014; Rusu *et al.*, 2009, 2008; Körtgen *et al.*, 2003; Tombari *et al.*, 2010; Guo *et al.*, 2013). However, in cryo-electron microscopy (cryo-EM), the voxel values in density maps represent the integration of density functions associated with atoms (Terashi and Kihara, 2018; Terwilliger *et al.*, 2020). Therefore, it is necessary to consider density vectors as an alternative to surface normals for describing points. CryoAlign utilizes mean shift equation to identify the direction of maximum density change around a single point, and assigns it as the density vector of the point. The unit vector is computed for each grid point $x_i (i = 1, ..., N)$ with a density value that no less than author-recommended contour level. The direction $\frac{\overrightarrow{y_i - x_i}}{|y_i - x_i|}$

†These authors should be regarded as Joint First Authors; *All correspondence should be addressed to Xin Gao (xin.gao@kaust.edu.sa) and Renmin Han (hanrenmin@sdu.edu.cn).

of unit vector reflects the trend of density values around the grid point x_i, of which the y_i is calculated by the following formula:

$$y_i = \frac{\sum_{n=1}^{N} k(x_i - x_n)\Phi(x_n)x_n}{\sum_{n'=1}^{N} k(x_i - x'_n)\Phi(x'_n)}, \tag{1}$$

where $k(p)$ is a Gaussian kernel function and $\Phi(x_i)$ is the density value of the grid point x_i. The $k(p)$ adjusts the weighting coefficients according to the input distance p and a bandwidth σ:

$$k(p) = \exp(-1.5|\frac{p}{\sigma}|^2). \tag{2}$$

The following experiments were conducted on the global alignment dataset; the sampling interval was set to 5Å. Figure S1a illustrates the dot score distribution between corresponding points for surface normals or density vectors. It is important to note that these corresponding points are obtained through MM-align (Mukherjee and Zhang, 2009) superimposition, which is defined as the ground truth. The figure shows two prominent peaks at opposite ends of the surface normals curve, indicating a certain degree of consistency but a larger number of orientation pairs that are completely opposite. Additionally, as the radius increases, the peak in the [0.75, 1.0] range continues to rise until it stops at a radius of 25Å. However, there are minimal changes observed in the other peak in the [-1.0, -0.75] range. The accuracy of estimated surface normals improves as more neighboring points are considered. Unfortunately, due to the differences in the imaging process, inconsistent results are inevitable with surface normals. In contrast, density vectors are computed based on the density value distribution, which reflects the imaging characteristics of the density maps. The much higher peak in the [0.75, 1.0] range suggests that density vectors exhibit better consistency compared to surface normals. Additionally, the larger bandwidth in the mean shift equation allows the algorithm to consider a larger range of neighboring points, similar to the radius parameter in surface normal estimation. Furthermore, to assess the descriptive capabilities of surface normals and density vectors, classical feature descriptors such as SHOT, PFH, and FPFH were selected, and their performances in feature matching were measured. Figure S1b displays the distribution of correct feature matching ratios, with dark lines representing density vector-based descriptors and shallow lines representing surface normal-based descriptors. The dark lines are positioned to the right of the shallow lines, indicating the stronger descriptive ability of density vectors.

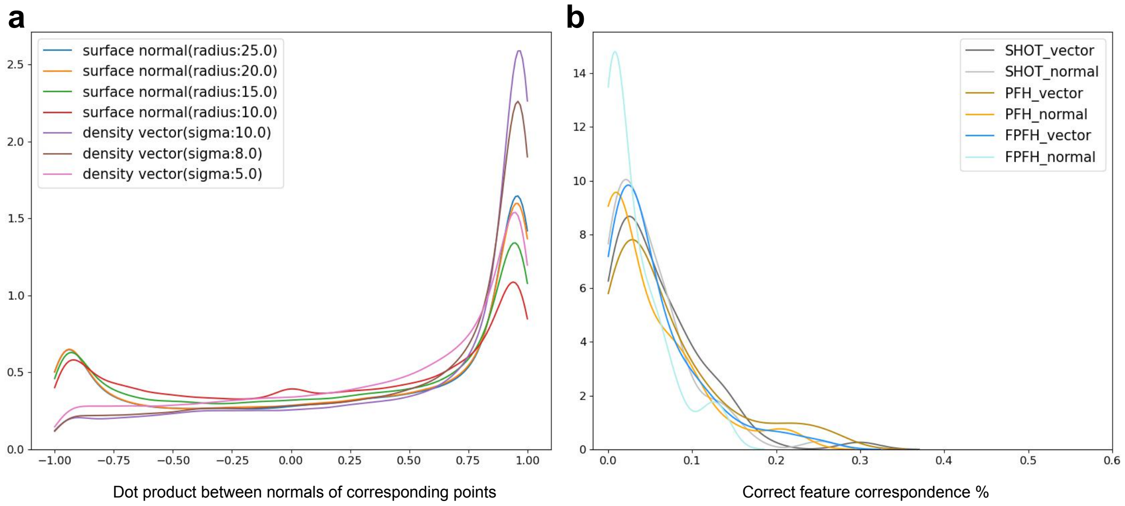

Figure S1: **Dot distribution of surface normals and density vectors. Noted that, this and following experiments were all conducted on the global alignment dataset (N=64).**

Existing point cloud descriptors typically encode the orientations of points in the local neighborhood into a feature vector using histogram statistics. Point correspondences are established by calculating the distance between feature vectors. However, due to the large number of original points, the feature matching process can be redundant and prone to errors. To address this, several 3D keypoint detectors have been proposed, including 3D SIFT (Lowe, 2004; Rusu and Cousins, 2011), 3D Harris (Sipiran and Bustos, 2011), and ISS (Zhong, 2009). The 3D Harris detector identifies corner points by considering the local spatial distribution. By analyzing the eigenvalues of the covariance matrix, the detector determines whether a point is located at a corner or edge. 3D SIFT extracts local features by analyzing the scale-space representation of the point cloud, utilizing gradient information and a histogram-based descriptor to characterize keypoints. ISS computes a unique signature for each point by analyzing its neighborhood in terms of surface curvature, surface normals, and other geometric properties. Figure S2 presents the number of extracted keypoints for different detectors. 3D Harris and ISS, being primarily based on point spatial distribution, yield a very limited number of keypoints. While a smaller quantity reduces computational complexity, it can also result in a loss of correct corresponding points. Moreover, these detectors do not consider the physical significance of density values, leading to an extremely uneven distribution of extracted keypoints. In CryoAlign, keypoints are identified by leveraging the fact that regions with high density values can indicate protein backbones. By combining mean shift and DBSCAN algorithms (Ester *et al.*, 1996), CryoAlign effectively identifies local dense points as keypoints. The initial

dense point is computed by the convergent result of the following iteration:

$$y_i^{t+1} = \frac{\sum_{n=1}^{N} k(y_i^t - x_n)\Phi(x_n)x_n}{\sum_{n'=1}^{N} k(y_i^t - x'_n)\Phi(x'_n)}. \tag{3}$$

Once the iteration converges in CryoAlign, the DBSCAN algorithm is employed to group the stable points y_i^n. DBSCAN merges adjacent points whose distance is closer than a given threshold (5.0Å in the experiments), forming clusters. These cluster centers are then considered density-based keypoints. Compared to existing detectors, CryoAlign's keypoints are distributed throughout the volume. In Figure S3, 3D Harris and ISS extract sparse keypoints, often resulting in only one or two points in a large region, as shown in the enlarged red boxes. 3D SIFT generates more keypoints; however, in the enlarged region, they are primarily located on the surface of the volume and lack an adequate number of neighboring points. In contrast, CryoAlign's extracted keypoints are distributed inside the volume, roughly representing the protein's backbones and having sufficient neighbors.

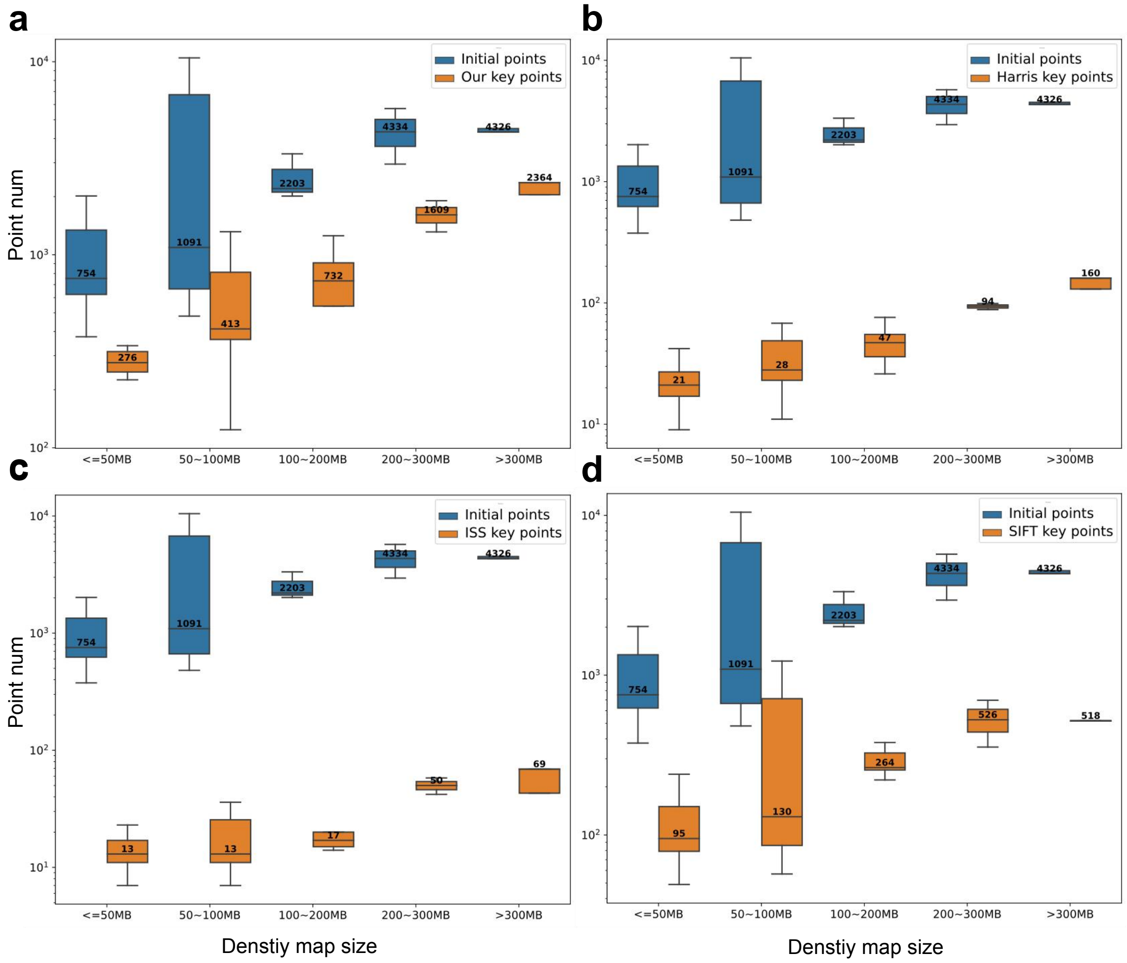

Figure S2: **The number of comparative keypoint detectors.** For map size groups "≤50MB", "50~100MB", "100~200MB", "200~300MB" and ">300MB", the sample sizes N=27, 17, 54, 80 and 15. The center, lower and upper lines in each box indicate the median, the first quartile and the third quartile, respectively. The number inside each box refers to the mean value. The whiskers show the 2.5% and 97.5% quantiles.

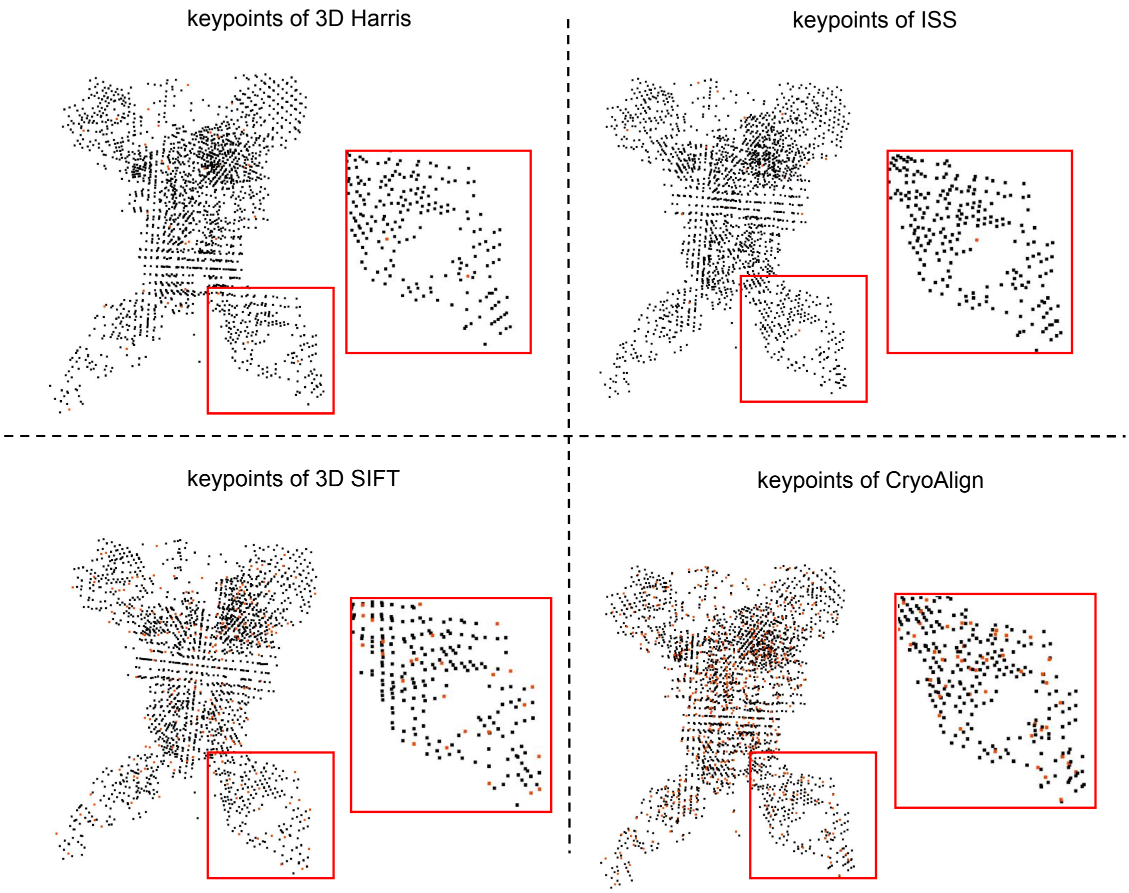

Figure S3: **An example (EMD-8789) with different extracted keypoints.**

Keypoints are often associated with descriptors to evaluate the structure extraction performance. To provide a comprehensive comparison, we selected existing commonly used point cloud descriptors: geometric attribute histogram based descriptors, PFH (Rusu *et al.*, 2008), FPFH (Rusu *et al.*, 2009) and SHOT (Salti *et al.*, 2014); spatial distribution histogram based descriptors 3DSC (Körtgen *et al.*, 2003), USC (Tombari *et al.*, 2010) and ROPS (Guo *et al.*, 2013). Figure S4 presents the distribution of correct feature correspondence ratios for different combinations of detectors and descriptors. The feature matching is directly performed by calculating the nearest neighbors in the feature domain. The accuracy of feature matching based on spatial distribution histogram descriptors, such as 3DSC, USC, and ROPS, is mainly concentrated in the interval [0, 0.1]. This indicates their limited applicability in density maps, as they heavily rely on statistics of point numbers in divided bins, resulting in poor representations of local structures. In contrast, SHOT and PFH perform the best among the methods, while FPFH is slightly inferior. Geometric attribute histogram-based descriptors, which utilize "normals" (in this case, density vectors), effectively capture the intrinsic

trends of density value changes. Considering the combination with detectors, we find that our clustering-based key-point extraction method consistently outperforms other methods. The distribution of our method, indicated by the red line, is generally located to the right of other curves, showing a higher correct ratio of feature matching. Table S1 calculates the average precision and recall of comparative methods. The combination of the SHOT descriptor with our clustering-based detector performs the best, achieving 16.8% matching accuracy.

Table S1: Average precision and recall of comparative methods

	Precision				Recall			
	Harris	ISS	SIFT	Our	Harris	ISS	SIFT	Our
SHOT	0.086	0.100	0.079	**0.168**	0.222	**0.259**	0.075	0.042
PFH	0.053	0.078	0.045	**0.145**	0.342	**0.439**	0.108	0.066
FPFH	0.040	0.062	0.033	**0.102**	0.258	**0.346**	0.091	0.055
3DSC	0.039	0.024	0.033	**0.069**	0.074	**0.088**	0.011	0.012
USC	0.024	0.015	0.027	**0.053**	0.024	**0.039**	0.013	0.004
ROPS	0.004	**0.027**	0.005	0.018	**0.038**	0.012	0.017	0.012

There are two metrics calculated in the feature matching, average precision and recall. For precision, the larger value denotes more accurate correspondences; for recall, the larger value indicates a higher key point utilization. In the tables of this supplementary material, for better presentation, the best results are marked in bold and the second best ones are underlined.

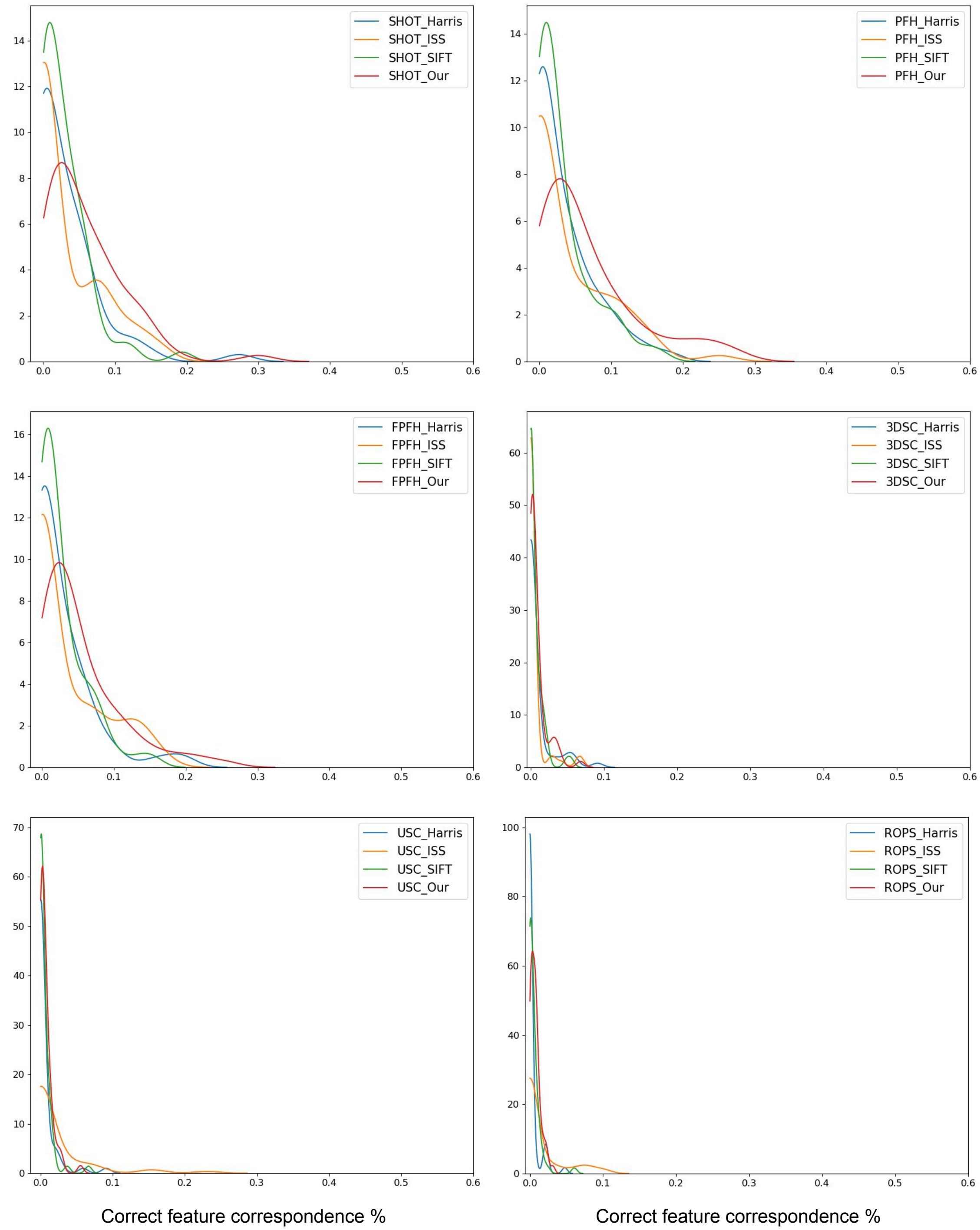

Figure S4: **The distribution of feature matching accuracy.**

Relying solely on the nearest neighbor calculation for feature matching can lead to insufficient accuracy. Even with SHOT or PFH, the correct ratio of feature matching is mainly concentrated approximately 10%. In CryoAlign, we utilize a bidirectional nearest point matching strategy to refine the feature correspondences further. Figure S5

demonstrates the distribution of correct feature correspondence ratios for different combinations in the mutual feature matching strategy. Compared to the direct nearest neighbor calculation, bidirectional feature matching exhibits a significant improvement in accuracy. For spatial distribution histogram-based methods such as 3DSC and USC, the distribution curves become flatter, and not all ratios fall within the [0, 0.1] interval. For geometric attribute histogram-based methods such as SHOT, PFH, and FPFH, the concentrated areas of the distribution curves shift from the interval [0.05, 0.15] to [0.1, 0.3]. By employing the mutual feature matching approach, we can also evaluate the average precision and recall, as shown in Table S2. The bidirectional nearest point matching filters out a large number of candidates, which sometimes results in insufficient remaining corresponding points to calculate rigid transformation parameters (at least 4 point pairs). Table S2 additionally provides the failure proportion for each comparative combination. 3D Harris and ISS consistently exhibit extremely high failure rates due to their inadequate number of identified keypoints. Conversely, 3D SIFT and our method extract enough candidate keypoints, resulting in a lower failure rate. Similar to the results in Table S1, the combination of SHOT and our method achieves the best balance between accuracy and success rate.

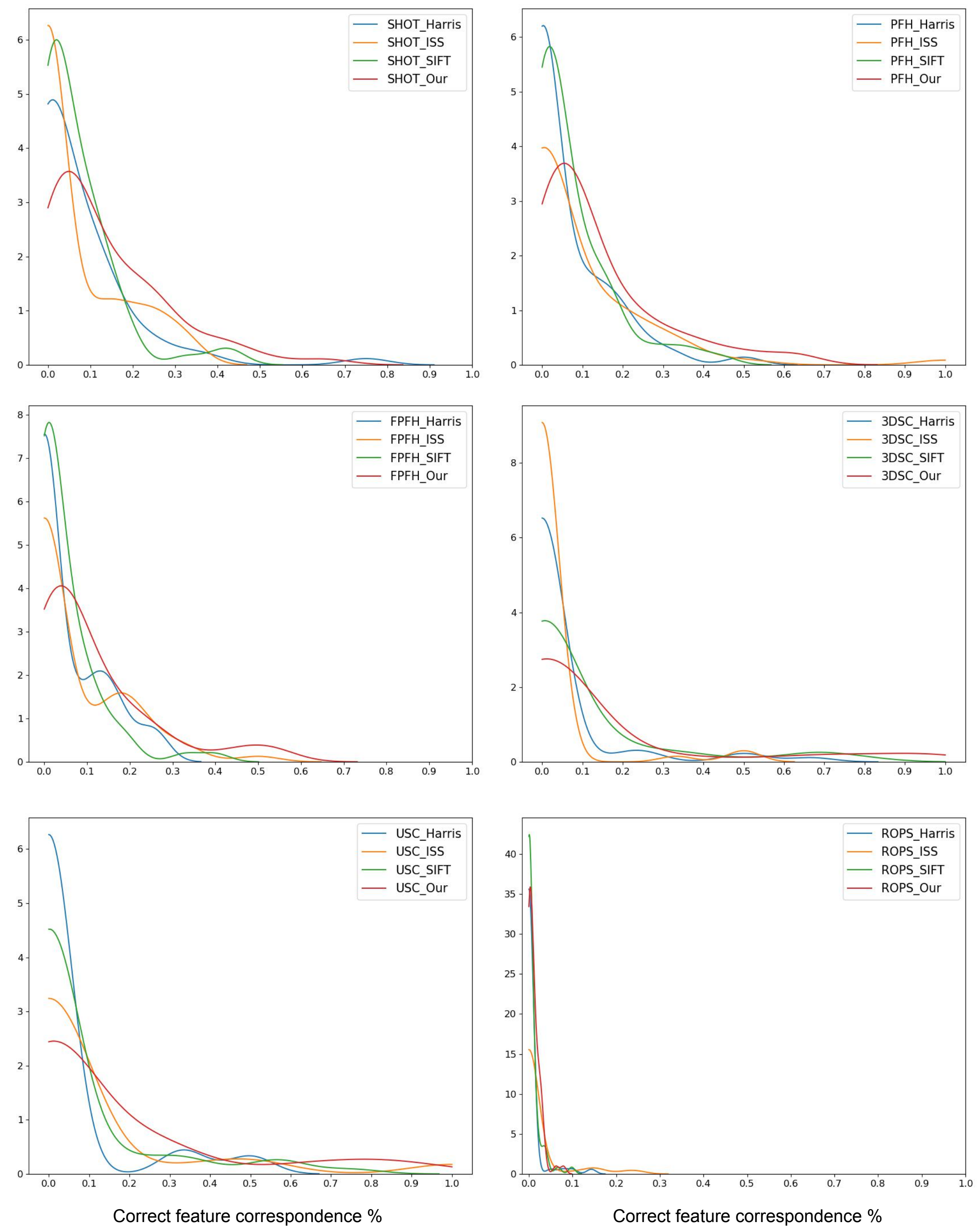

Figure S5: **The distribution of feature matching accuracy in mutual feature matching strategy.**

Table S2: Average precision and recall of comparative methods in mutual feature matching

	Precision (Failure)				Recall			
	Harris	ISS	SIFT	Our	Harris	ISS	SIFT	Our
SHOT	0.213(68.75%)	**0.298**(89.06%)	0.148(14.06%)	0.292(**0.0%**)	0.280	**0.335**	0.049	0.027
PFH	0.099(37.5%)	0.170(65.6%)	0.103(9.37%)	**0.224**(**0.0%**)	0.229	**0.389**	0.061	0.023
FPFH	0.088(35.94%)	0.122(59.4%)	0.060(7.82%)	**0.178**(**0.0%**)	0.230	**0.308**	0.044	0.021
3DSC	0.250(98.4%)	**0.500**(98.4%)	0.267(79.7%)	0.291(**60.94%**)	0.100	**0.500**	0.035	0.008
USC	-(100.0%)	-(100.0%)	0.311(87.5%)	**0.334**(**73.44%**)	-	-	**0.027**	0.007
ROPS	0.002(34.38%)	**0.027**(56.25%)	0.008(**0.0%**)	0.023(**0.0%**)	0.003	**0.049**	0.009	0.004

There are three metrics calculated in the feature matching, average precision, failure ratio and recall. For precision, the larger value denotes more accurate correspondences; for failure ratio, the smaller value indicates higher stability; for recall, the larger value indicates a higher key point utilization.

S2 Density based SHOT descriptor calculation

The SHOT descriptor starts with a local reference frame (RF) calculation, which refers to a coordinate system defined locally for each point in the point cloud. The local RF is invariant to translations and rotations and robust to noise and clutter, similar to rotation and/or scale invariance injected into 2D descriptors. The RF determination actually involves the assignment of three coordinate axes X, Y and Z. Given a clustering based key-point $\mathbf{P}_{key}$, SHOT first builds a covariance matrix $\mathbf{M}$ by collecting neighbor points within the radius R:

$$\mathbf{M} = \frac{1}{\sum_{i:d_i \leq R}(R - d_i)} \sum_{i:d_i \leq R} (R - d_i)(\mathbf{P_i} - \mathbf{P}_{key})(\mathbf{P_i} - \mathbf{P}_{key})^T. \quad (4)$$

Then, SHOT refers to the three unit eigenvectors in decreasing eigenvalue order as the $\mathbf{x}^+$; $\mathbf{y}^+$ and $\mathbf{z}^+$ axes. With $\mathbf{x}^-$; $\mathbf{y}^-$ and $\mathbf{z}^-$, SHOT denotes instead the opposite unit vectors. Finally, the disambiguated $\mathbf{x}$ axis is defined as :

$$S_x^+ \doteq \{i : d_i \leq R \wedge (\mathbf{P_i} - \mathbf{P}_{key}) \cdot \mathbf{x}^+ \geq 0\}, \tag{5}$$

$$S_x^- \doteq \{i : d_i \leq R \wedge (\mathbf{P_i} - \mathbf{P}_{key}) \cdot \mathbf{x}^- > 0\}, \tag{6}$$

$$\tilde{S}_x^+ \doteq \{i : i \in M(k) \wedge (\mathbf{P_i} - \mathbf{P}_{key}) \cdot \mathbf{x}^+ \geq 0\}, \tag{7}$$

$$\tilde{S}_x^- \doteq \{i : i \in M(k) \wedge (\mathbf{P_i} - \mathbf{P}_{key}) \cdot \mathbf{x}^- > 0\}, \tag{8}$$

$$\mathbf{x} = \begin{cases} \mathbf{x}^+, |S_x^+| > |S_x^-| \\ \mathbf{x}^-, |S_x^+| < |S_x^-| \\ \mathbf{x}^+, |S_x^+| = |S_x^-| \wedge |\tilde{S}_x^+| > |\tilde{S}_x^-| \\ \mathbf{x}^-, |S_x^+| = |S_x^-| \wedge |\tilde{S}_x^+| < |\tilde{S}_x^-|, \end{cases}, \tag{9}$$

where $M(k)$ is the subset of points within the support whose distance from the feature point is among the k closest to the median distance. The same procedure is used to disambiguate the $\mathbf{z}$ axis and the $mathbfy$ axis is obtained as $\mathbf{z} \times \mathbf{x}$.

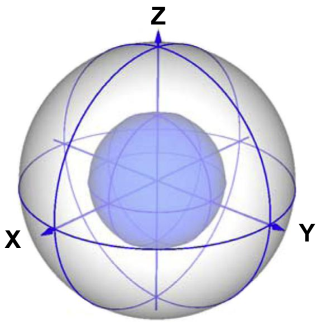

Figure S6: **Signature structure of SHOT.**

After establishing the RF, the geometry information, such as density vectors, is encoded by partitioning bins to represent local features. In the case of the SHOT descriptor, the spherical support area is divided into 32 small volumes based on two radial divisions (inner and outer balls), two elevation divisions (northern and southern hemispheres), and eight azimuth divisions. However, to enhance clarity, only four azimuth divisions are plotted in Figure S6. For each volume, the histogram is built by accumulating points into bins according to the cosine between the density vector at the neighbor point P_i and the local $\mathbf{z}$ axis at the query key-point $\mathbf{P}_{key}$. To alleviate boundary effects, for each point being accumulated into a specific local histogram bin, SHOT performs quadrilinear interpolation with its neighbors, i.e. the

neighboring bin in the local histogram and the bins having the same index in the local histograms corresponding to the neighboring subdivisions of the grid. Figure S7 provides a graphic description of the quadrilinear interpolation process. It shows how each point contributes to multiple bins by assigning different weights based on the interpolation scheme. In CryoAlign, the number of bins for the local histogram is set to 11, and the length of the density-based SHOT descriptor is 32×11 (352).

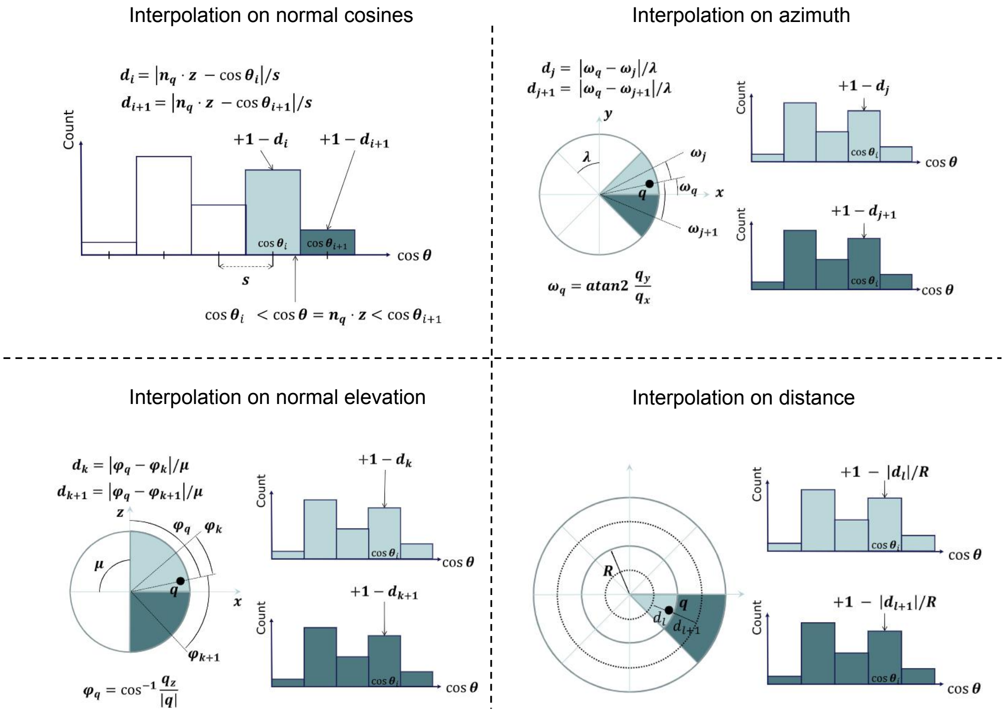

Figure S7: **Quadrilinear interpolation to accumulate weights into histograms.**

S3 Application in atomic model fitting

S3.1 Ranked results in atomic model fitting

Many macromolecular complexes have multiple protein chains with very similar shapes. The precise alignment of a specific single chain which has similar "brother" chains raises a challenge to rank the exact transformation as high as possible in a number of alternative solutions. In the main text, we provide ranking information based on the optimal matching results. In this section, we enumerate the remaining alignments according to the ranking information and subsequently calculate RMSD values by determining the most suitable chain in Figure S8. It is evident that all candidate chains exhibit high structural similarity, nearly identical. The alignment of a single chain actually corresponds to

multiple optimal parameters, the main reason for the low rankings of correct alignment. However, besides those optimal alignments, VESPER may provide multiple sub-optimal alignment results for the same target region, as observed in the rank#3 and rank#4 results of chain A. The consistent relative low accuracy of VESPER assigns these specious alignment results approximate similarity scores and similar rankings. If the precision of correct alignment cannot stand out, these results may significantly affect its ranking. Compared to VESPER, CryoAlign delivers more accurate superimposition, with the RMSD values around 1.3Å. This high precision ensures the non-existence of sub-optimal alignments for the same target region in the top-ranking results.

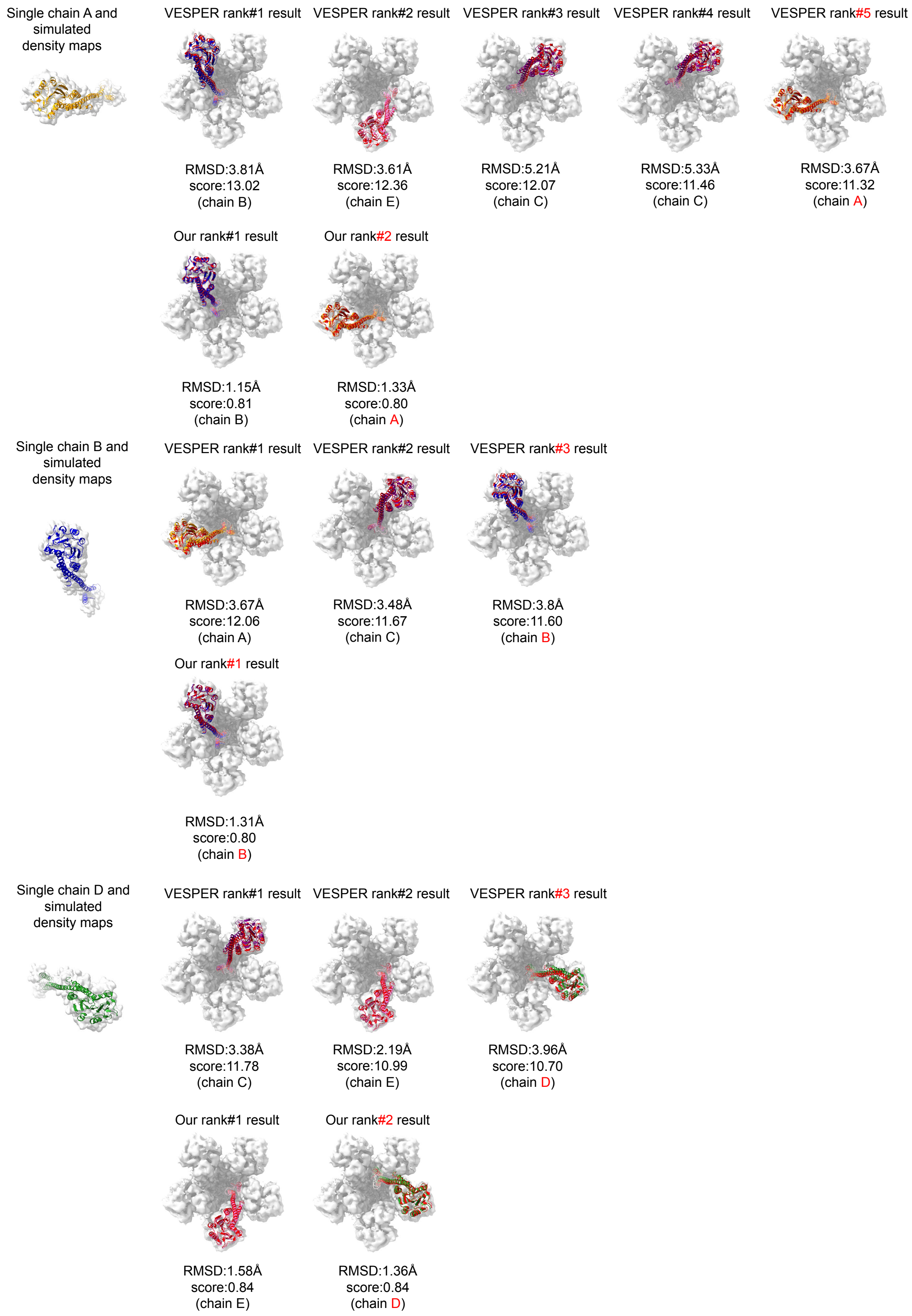

Figure S8: **Ranked alignment results of the first example in atomic model fitting.** The RMSD values are calculated with the most matching chain, shown within parentheses. The query chain and corresponding alignment are colored red.

S3.2 More atomic model fitting results

We have collected more atomic model fitting results in Table S3, which provides alignment accuracy for each single chain. The RMSD values ranging from 30 to 60Å clearly demonstrate the inadequacy of gmfit and fitmap without manual intervention. VESPER, on the other hand, is capable of identifying the correct superimposition from the top ten candidates. The optimal alignment in VESPER is calculated by an exhaustive search with a given rotation interval (which is usually set to 10 degrees). Such a rough grid searching usually makes VESPER neglect the very detailed structural differences of the compared maps, and results in a sub-optimal alignment, especially for candidate chains exhibiting structural similarity in rotation. Consequently, the similarity score of correct alignment fails to stand out among top-ranked results, leading to a low ranking. In the case of chain E in EMD-0440 and chain D in EMD-4400, the acceptable alignment is not even present in the top ten list. In comparison, CryoAlign achieves higher alignment accuracy, as indicated by lower RMSD values. Moreover, the correct parameters tend to rank higher in CryoAlign's candidate lists, further highlighting the superiority of the feature-based alignment approach.

Table S3: Alignment evaluation in atomic model fitting

Protein complex	chain	CryoAlign/Å	VESPER/Å	gmfit/Å	fitmap/Å
emd-0287(6hv8.cif)	A	**0.94**	3.56	38.46	54.11
	B	**0.67**	3.68	50.83	46.82
emd-0346(6h52.cif)	A	**0.47**	2.46	75.59	0.96
	B	**0.50**	2.58	94.86	1.27
emd-0440(6nd1.cif)	A	**0.41**	2.72	36.65	67.09
	B	**0.76**	3.42	33.21	50.99
	E	**0.82**	43.93	55.35	76.62
emd-0868(6lba.cif)	A	**1.04**	1.54	46.08	31.85
	B	**0.59**	3.70(#9)	44.77	69.05
	C	**0.48**	58.71	38.55	69.59
	D	**0.76**	2.62	45.85	73.73
emd-3340(5fwp.cif)	A	**1.35**	1.92	63.25	63.92
	B	**2.01**(#5)	4.11(#5)	21.55	41.89
	K	**4.47**	5.00	37.19	26.71
emd-3605(5n9y.cif)	A	**1.33**(#2)	3.67(#5)	62.15	85.00
	B	**1.31**	3.80(#3)	60.28	74.14
	C	**0.41**	3.66	31.23	73.52
	D	**1.36**(#2)	3.96(#3)	61.06	79.36
	E	**0.98**	2.06	32.02	8.05
emd-3861(5oyg.cif)	A	**0.48**	4.39	53.62	61.33
	B	**0.27**	3.86	46.85	55.69
emd-4400(6i2t.cif)	A	**1.27**(#3)	2.32	63.52	75.85
	B	**1.03**	2.30(#2)	63.57	42.64
	C	**1.03**	3.96(#2)	35.02	80.75
	D	**0.95**	73.82	59.62	112.40
	J	**4.39**	9.29	65.23	53.25

S3.3 Extended results in flexible fitting

With the help of uniform sampling and structure-based clustering, the point cloud representation effectively bridges the gap between atoms and density voxels. Given the accurate rigid alignment, the flexible fitting, also called non-rigid registration in the point cloud, can be easily estimated by considering the displacements for matched point pairs. Many methods (Zampogiannis *et al.*, 2019; Hirose, 2021; Zheng and Doermann, 2006; Ma *et al.*, 2018; Zhang *et al.*, 2018) have been proposed for flexible fitting. In CryoAlign, we select the Bayesian coherent point drift algorithm (Hirose, 2021) to estimate displacements for each point. To illustrate the performance, we considered two conformations of the heterodimeric ABC exporter TmrAB (EMD-4775, EMD-4776). Fixing EMD-4776 (PDB ID: 6rai, [http://doi.org/10.2210/pdb6rai/pdb]) as the reference map, we extracted chains A and B from EMD-4775 (PDB ID: 6rah, [http://doi.org/10.2210/pdb6rah/pdb]) as assembling candidates, as depicted in Figure S9a, where the colored point clouds are attached nearby. Fitmap, lacking initial poses from experienced researchers, struggles to assemble the two chains properly. Both CryoAlign and VESPER place the chains into the appropriate positions according to accurate local alignment. Then, these point clouds representing chains are transformed respectively and merged into a comprehensive and larger point cloud. The assembly of point clouds is an initial model representation of the protein structure (see Figure S9b). Fitmap provides a poor initial assembly model, making flexible fitting incorrectly reflect the molecular motion. In contrast, both CryoAlign and VESPER offer well-assembled point clouds, resulting in successful displacement estimation between matched points, as shown in Figure S9c. These point displacements can be conveniently applied to atomic models, as both points and atoms share the same coordinate system. Furthermore, compared to VESPER, CryoAlign achieves better fitting and lower RMSD, with an refinement of 0.1Å and 0.9Å for chains A and B, respectively. This is primarily because CryoAlign offers superior rigid alignment, thereby simplifying the establishment of point correspondences during the flexible fitting process. Notably, regions undergoing main conformational changes are magnified for enhanced visualization.

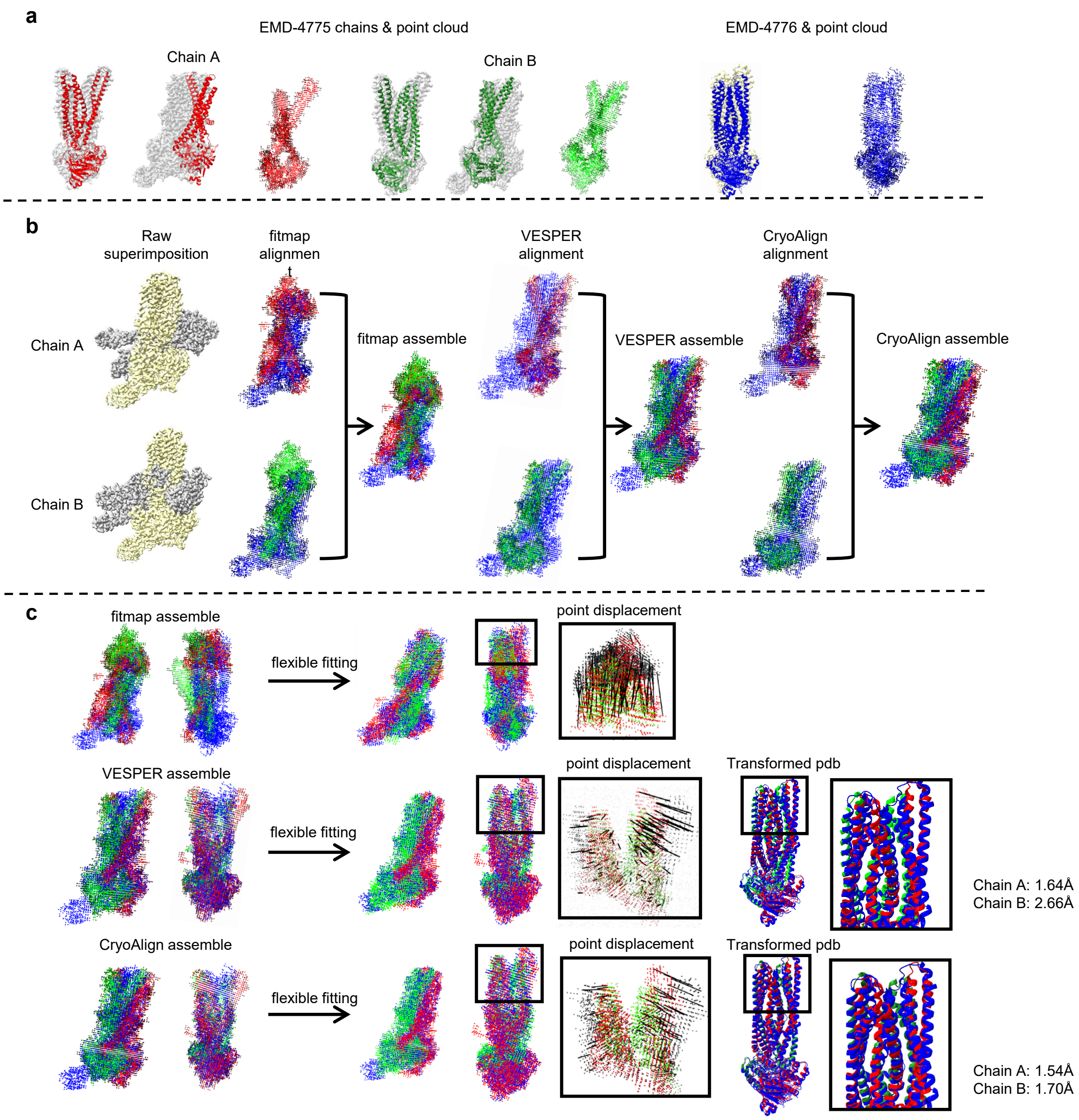

Figure S9: **An example for flexible transformation in atomic model fitting.** **a** The source map is EMD-4775 (PDB ID: 6rah, [http://doi.org/10.2210/pdb6rah/pdb]), whose two chains A and B are extracted. The target map is EMD-4776 (PDB ID: 6rai, [http://doi.org/10.2210/pdb6rai/pdb]). **b** The alignment results of individual chains and corresponding assembly results. **c** Based on the point clouds assembled by the compared methods, flexible fitting is applied to estimate displacements between matching points. Furthermore, the estimated displacement is performed on the atomic model to generate transformed PDB structure. The RMSD values are individually calculated for two chains.

In Figure S10, we present an additional example of the E. coli replicative DNA polymerase complex bound to DNA (EMD-3198, PDB ID: 5fkv, [http://doi.org/10.2210/pdb5fkv/pdb] and EMD-3201, PDB ID: 5fku, [http://doi.org/10.2210/pdb5fku/pdb]). The primary conformational distinction involves a large rotation in the chain A subunit, highlighted within the black

box. Based on the accurate assembly provided by CryoAlign, displacement construction between point pairs becomes straightforward. This transformation is further validated by the resulting PDB structure.

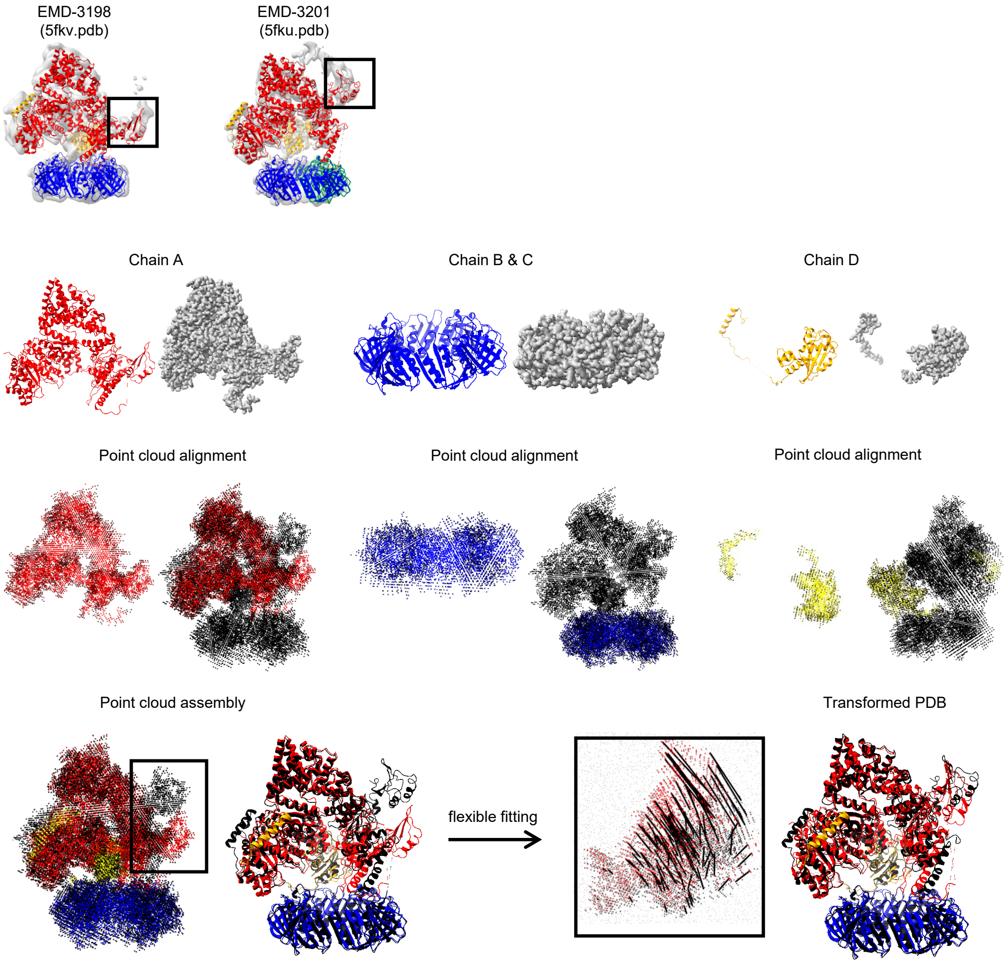

Figure S10: **Another example for flexible transformation in atomic model fitting.** The source map is EMD-3198 (PDB ID: 5fkv, [http://doi.org/10.2210/pdb5fkv/pdb]), whose chains A, B, C and D are extracted. The target map is EMD-3201 (PDB ID: 5fku, [http://doi.org/10.2210/pdb5fku/pdb]). All chains are individually aligned by CryoAlign to the target map and assembled into a larger complex. Furthermore, the point displacements are estimated by flexible fitting to transform the corresponding PDB structure. The subunit with a large rotation is precisely depicted.

S4 Initial mask localization

A rough initial position of the mask helps to reduce the search space of alignment parameters. To achieve efficient and accurate local alignment, users can employ the rough result of exhaustive methods as the initial state and use CryoAlign to further search the best alignment parameters by only a few masking operations around the initial superimposition.

Here, we test the alignment performance of the combination of the exhaustive method VESPER and CryoAlign. Table S4 summarizes the average RMSD and failure ratios for initial pose generation at different sampling intervals. The initial alignment results were calculated using the exhaustive search method VESPER, and the CryoAlign refinement results were acquired by searching $\pm r$ ranges around the initial ones. Due to the large sampling intervals, a failure threshold of 30Å is applied in this experiment. With intervals exceeding 10Å, even if the initial poses are imprecise, CryoAlign demonstrates the capability to rectify most of them by exploring the surrounding regions. Compared to the resolution range "<5Å" in the main text Table 4, a decrease in the failure ratio is evident, indicating that a well-positioned mask contributes to improved alignment success. This improvement is achieved by masking feature points of large volumes, which interfere with feature matching processes. However, CryoAlign may not fully exploit well-positioned initial poses within the straightforward spherical mask strategy, since rotation and translation statistically have little effect on the feature construction.

Table S4: Alignment evaluation with given initial pose in local alignment

	Sampling intervals for initial exhaustive			
Initial exhaustive	5Å	10Å	15Å	20Å
5Å	6.07/0%	10.26/6.56%	15.57/72.13%	7.76/94.26%
5.0~10.0Å	6.48/14.29%	7.71/14.29%	13.55/21.43%	19.45/78.57%
Cross res.	7.02/13.8%	11.10/23.08%	18.47/61.54%	21.01/90.77%
CryoAlign refinement				
5Å	4.34/5.74%	3.93/19.67%	3.99/21.31%	4.12/23.77%
5.0~10.0Å	2.89/7.14%	3.13/7.14%	3.42/7.14%	3.38/7.14%
Cross res.	5.20/13.8%	4.47/18.46%	4.77/21.53%	4.27/27.69%

S5 Parameter settings

In the generation of the initial point cloud, the primary parameter is the voxel sampling interval. In this study, we utilized 5Å as the default setting. This choice is suitable for density maps with resolution higher than 10Å, ensuring a sufficient number of points while maintaining acceptable execution times. Notably, if the input map represents a

single chain or has small volumes, the sampling interval is better to be adjusted to 2 ∼ 3Å for improved performance. It is crucial to ensure that the number of initial sampling points remains above 200. Then in the feature calculation and feature-based alignment processes, there are many parameters for the radius and weighting coefficients. We strongly recommend researchers follow the default settings, however for those requiring further details, interested readers can refer to Table S5.

Table S5: Parameters of CryoAlign.

	Parameters	Description
Density-based vector	Meanshift_sigma_1	Sigma of Gaussian filter, default=8.0.<br>The larger value is due to the only once calculation of the equation.
Key-point extraction	Meanshift_sigma_2	Sigma of Gaussian filter, default=3.0.<br>The smaller value is for the iteration process.
	DBSCAN_radius_1	Radius of the DBSCAN, default=3*Voxel.<br>Remove the outliers and island clusters.
	DBSCAN_radius_2	Radius of the DBSCAN, default=1*Voxel.<br>Find the clustering centers as the key-points.
Feature calculation	SHOT_radius	Radiuis of the feature descriptor, default=5*Voxl.<br>The larger value means considering the more neighboring points.
Alignment	Noise_bound	Threshold to determine the false matching points during feature matching, default=1*Voxel.
	ICP_points	Use key-points or initial points as the inputs for ICP algorithm, default=initial points.
	score_threshold	Threshold to determine the good matching based on density vectors, default=0.6.

References

Ester, M., Kriegel, H.-P., Sander, J., Xu, X., *et al.* (1996). A density-based algorithm for discovering clusters in large spatial databases with noise. In *kdd*, volume 96, pages 226–231.

Guo, Y., Sohel, F., Bennamoun, M., Lu, M., and Wan, J. (2013). Rotational projection statistics for 3d local surface description and object recognition. *International journal of computer vision*, **105**, 63–86.

Hirose, O. (2021). A bayesian formulation of coherent point drift. *IEEE Transactions on Pattern Analysis and Machine Intelligence*, **43**(7), 2269–2286.

Körtgen, M., Park, G.-J., Novotni, M., and Klein, R. (2003). 3d shape matching with 3d shape contexts. In *The 7th central European seminar on computer graphics*, volume 3, pages 5–17. Budmerice Slovakia.

Lowe, D. G. (2004). Distinctive image features from scale-invariant keypoints. *International journal of computer vision*, **60**, 91–110.

Ma, J., Wu, J., Zhao, J., Jiang, J., Zhou, H., and Sheng, Q. Z. (2018). Nonrigid point set registration with robust transformation learning under manifold regularization. *IEEE transactions on neural networks and learning systems*, **30**(12), 3584–3597.

Mukherjee, S. and Zhang, Y. (2009). Mm-align: a quick algorithm for aligning multiple-chain protein complex structures using iterative dynamic programming. *Nucleic acids research*, **37**(11), e83–e83.

Rusu, R. B. and Cousins, S. (2011). 3d is here: Point cloud library (pcl). In *2011 IEEE international conference on robotics and automation*, pages 1–4. IEEE.

Rusu, R. B., Blodow, N., Marton, Z. C., and Beetz, M. (2008). Aligning point cloud views using persistent feature histograms. In *2008 IEEE/RSJ international conference on intelligent robots and systems*, pages 3384–3391. IEEE.

Rusu, R. B., Blodow, N., and Beetz, M. (2009). Fast point feature histograms (fpfh) for 3d registration. In *2009 IEEE international conference on robotics and automation*, pages 3212–3217. IEEE.

Salti, S., Tombari, F., and Di Stefano, L. (2014). Shot: Unique signatures of histograms for surface and texture description. *Computer Vision and Image Understanding*, **125**, 251–264.

Sipiran, I. and Bustos, B. (2011). Harris 3d: a robust extension of the harris operator for interest point detection on 3d meshes. *The Visual Computer*, **27**, 963–976.

Terashi, G. and Kihara, D. (2018). De novo main-chain modeling for em maps using mainmast. *Nature communications*, **9**(1), 1618.

Terwilliger, T. C., Adams, P. D., Afonine, P. V., and Sobolev, O. V. (2020). Cryo-em map interpretation and protein model-building using iterative map segmentation. *Protein science*, **29**(1), 87–99.

Tombari, F., Salti, S., and Di Stefano, L. (2010). Unique shape context for 3d data description. In *Proceedings of the ACM workshop on 3D object retrieval*, pages 57–62.

Zampogiannis, K., Fermüller, C., and Aloimonos, Y. (2019). Topology-aware non-rigid point cloud registration. *IEEE Transactions on Pattern Analysis and Machine Intelligence*, **43**(3), 1056–1069.

Zhang, S., Yang, K., Yang, Y., Luo, Y., and Wei, Z. (2018). Non-rigid point set registration using dual-feature finite mixture model and global-local structural preservation. *Pattern Recognition*, **80**, 183–195.

Zheng, Y. and Doermann, D. (2006). Robust point matching for nonrigid shapes by preserving local neighborhood structures. *IEEE transactions on pattern analysis and machine intelligence*, **28**(4), 643–649.

Zhong, Y. (2009). Intrinsic shape signatures: A shape descriptor for 3d object recognition. In *2009 IEEE 12th international conference on computer vision workshops, ICCV workshops*, pages 689–696. IEEE.
