# Supplementary material for: Accurate global and local 3D alignment of cryo-EM density maps using local spatial structural features
Source: Nat Commun. 2024 Feb 21;15:1593. doi: 10.1038/s41467-024-45861-4 (PMC10881975; doi:10.1038/s41467-024-45861-4)
Supplement: Supplementary file 3 — Reporting Summary [file 41467_2024_45861_MOESM3_ESM.pdf]

nature portfolio

Corresponding author(s): Xin Gao, Renmin Han

Last updated by author(s): Jan 19, 2024

Reporting Summary

Nature Portfolio wishes to improve the reproducibility of the work that we publish. This form provides structure for consistency and transparency in reporting. For further information on Nature Portfolio policies, see our Editorial Policies and the Editorial Policy Checklist.

Statistics

For all statistical analyses, confirm that the following items are present in the figure legend, table legend, main text, or Methods section.

n/a	Confirmed	
☐	☒	The exact sample size (*n*) for each experimental group/condition, given as a discrete number and unit of measurement
☒	☐	A statement on whether measurements were taken from distinct samples or whether the same sample was measured repeatedly
☒	☐	The statistical test(s) used AND whether they are one- or two-sided<br>*Only common tests should be described solely by name; describe more complex techniques in the Methods section.*
☒	☐	A description of all covariates tested
☒	☐	A description of any assumptions or corrections, such as tests of normality and adjustment for multiple comparisons
☐	☒	A full description of the statistical parameters including central tendency (e.g. means) or other basic estimates (e.g. regression coefficient) AND variation (e.g. standard deviation) or associated estimates of uncertainty (e.g. confidence intervals)
☒	☐	For null hypothesis testing, the test statistic (e.g. *F*, *t*, *r*) with confidence intervals, effect sizes, degrees of freedom and *P* value noted<br>*Give P values as exact values whenever suitable.*
☒	☐	For Bayesian analysis, information on the choice of priors and Markov chain Monte Carlo settings
☒	☐	For hierarchical and complex designs, identification of the appropriate level for tests and full reporting of outcomes
☒	☐	Estimates of effect sizes (e.g. Cohen's *d*, Pearson's *r*), indicating how they were calculated

Our web collection on statistics for biologists contains articles on many of the points above.

Software and code

Policy information about availability of computer code

Data collection	All data used is from the public EMDB and PDB data repositories. No software was used for data collection.
Data analysis	The method CryoAlign is freely available via https://github.com/HeracleBT/CryoAlign. The visualization of density maps and PDB atomic models was performed in UCSF ChimeraX (version: 1.7). For the illustration, an example dataset and corresponding code are available in https://github.com/HeracleBT/CryoAlign/tree/main/data/example_dataset.

For manuscripts utilizing custom algorithms or software that are central to the research but not yet described in published literature, software must be made available to editors and reviewers. We strongly encourage code deposition in a community repository (e.g. GitHub). See the Nature Portfolio guidelines for submitting code & software for further information.

Data

Policy information about availability of data

All manuscripts must include a data availability statement. This statement should provide the following information, where applicable:

- Accession codes, unique identifiers, or web links for publicly available datasets
- A description of any restrictions on data availability
- For clinical datasets or third party data, please ensure that the statement adheres to our policy

The datasets of cryo-EM maps for global and local alignment are provided in Source Data Files (global_alignment.xlsx, local_alignment.xlsx, supplementary material

data.xlsx), involving accession codes and web links. The cryo-EM maps and fitted PDB entries can be downloaded from EMDB and PDB, respectively. For the illustration, an example dataset and corresponding code are available in https://github.com/HeracleBT/CryoAlign/tree/main/data/example_dataset.

Research involving human participants, their data, or biological material

Policy information about studies with human participants or human data. See also policy information about sex, gender (identity/presentation), and sexual orientation and race, ethnicity and racism.

Reporting on sex and gender	N/A
Reporting on race, ethnicity, or other socially relevant groupings	N/A
Population characteristics	N/A
Recruitment	N/A
Ethics oversight	N/A

Note that full information on the approval of the study protocol must also be provided in the manuscript.

Field-specific reporting

Please select the one below that is the best fit for your research. If you are not sure, read the appropriate sections before making your selection.

☒ Life sciences ☐ Behavioural & social sciences ☐ Ecological, evolutionary & environmental sciences

For a reference copy of the document with all sections, see nature.com/documents/nr-reporting-summary-flat.pdf

Life sciences study design

All studies must disclose on these points even when the disclosure is negative.

Sample size	All raw data we used is from two public databases, EMDB and PDB. The selecting entries were filtered from open source datasets provided by existing works, and the process is described at the section “Datasets of density maps and metrics”. For example, both global and local alignment datasets are from https://kiharalab.org/vesper_data. The atomic modeling dataset is from https://www.nature.com/articles/s41467-022-31748-9#data-availability. The global alignment dataset consists of 33 high-resolution (<5Å) density maps and 23 intermediate-resolution (5-10Å) maps. These maps were paired to 64 inputs following the group identifications (provided by VESPER datasets). The local alignment dataset consists of 14 high-resolution density maps and 10 intermediate-resolution maps. These maps were also followed the group identifications to generate 201 paired inputs. The sizes of testing dataset ensure the statistical significance of performance evaluation with comparative approaches.
Data exclusions	The maps without fitted PDB entries and maps of resolution lower than 10 Å were excluded. The reason is that our method aims to process the maps with high resolution, and the evaluation of alignment accuracy needs PDB structures.
Replication	The results can be replicated using the same datasets provided in Supplementary data. If all attempts at replication were successful. Our code is open-source on Github (https://github.com/HeracleBT/CryoAlign), and the reproducibility can be verified.
Randomization	Randomization was not relevant to our study because this type of study does not use group allocation.
Blinding	Blinding was not relevant to our study because this type of study does not use group allocation.

Reporting for specific materials, systems and methods

We require information from authors about some types of materials, experimental systems and methods used in many studies. Here, indicate whether each material, system or method listed is relevant to your study. If you are not sure if a list item applies to your research, read the appropriate section before selecting a response.

Materials & experimental systems

n/a	Involved in the study
☒	☐ Antibodies
☒	☐ Eukaryotic cell lines
☒	☐ Palaeontology and archaeology
☒	☐ Animals and other organisms
☒	☐ Clinical data
☒	☐ Dual use research of concern
☒	☐ Plants

Methods

n/a	Involved in the study
☒	☐ ChIP-seq
☒	☐ Flow cytometry
☒	☐ MRI-based neuroimaging

Plants

Seed stocks

Report on the source of all seed stocks or other plant material used. If applicable, state the seed stock centre and catalogue number. If plant specimens were collected from the field, describe the collection location, date and sampling procedures.

Novel plant genotypes

Describe the methods by which all novel plant genotypes were produced. This includes those generated by transgenic approaches, gene editing, chemical/radiation-based mutagenesis and hybridization. For transgenic lines, describe the transformation method, the number of independent lines analyzed and the generation upon which experiments were performed. For gene-edited lines, describe the editor used, the endogenous sequence targeted for editing, the targeting guide RNA sequence (if applicable) and how the editor was applied.

Authentication

Describe any authentication procedures for each seed stock used or novel genotype generated. Describe any experiments used to assess the effect of a mutation and, where applicable, how potential secondary effects (e.g. second site T-DNA insertions, mosiacism, off-target gene editing) were examined.
